# Optimising Autologous Breast Reconstruction: Pneumothorax and Pneumomediastinum—A Literature Review and First Reported Case of Pneumomediastinum Following Bilateral DIEP Flap Reconstruction

**DOI:** 10.3390/jpm16090458

**Published:** 2026-08-30

**Authors:** Akshay Soni, Ishith Seth, Kaiyang Lim, Alexander Phan, Richard J. Ross, Mathew Lee, Warren M. Rozen

**Affiliations:** 1 Department of Plastic and Reconstructive Surgery, Peninsula University Hospital, Bayside Health, Frankston, VIC 3199, Australia; 2 Faculty of Medicine and Surgery, Central Clinical School, Monash University, Melbourne, VIC 3004, Australia

**Keywords:** pneumothorax, pneumomediastinum, autologous breast reconstruction, DIEP flap, postoperative complications, thoracic injury

## Abstract

**Background**: Thoracic air complications after autologous breast reconstruction (ABR) are uncommon and incompletely characterised. A personalised perioperative approach requires integration of patient anatomy, reconstructive technique, anaesthetic exposures and the postoperative clinical trajectory rather than reliance on population-level estimates alone. **Methods**: We report a 41-year-old woman in whom pneumomediastinum was identified after bilateral DIEP flap reconstruction and performed a focused literature review restricted to primary studies of pneumothorax or pneumomediastinum following autologous flap breast reconstruction. Searches across MEDLINE-Ovid, PubMed and Wiley Online Library using predefined MeSH terms were conducted. Data extraction focused on study characteristics, incidence, mechanism of injury, perioperative factors, and management. **Results**: The patient developed postoperative hypotension and persistent tachycardia, followed by pleuritic chest pain. CT excluded pulmonary embolism and oesophageal perforation but demonstrated Moderate volume pneumomediastinum without pneumothorax. She was managed conservatively and discharged on postoperative day 7. Five primary studies met the eligibility criteria. Study-specific pneumothorax frequencies were 1/463 (0.22%) after rib-sparing free-flap reconstruction, 3/749 (0.4%) after extended latissimus dorsi reconstruction, and 4/180 patients (2.2%; 1.4 per 100 internal mammary vessel dissections). Reported mechanisms included pleural injury during internal mammary vessel dissection, inadvertent puncture during regional anaesthesia, barotrauma under positive-pressure ventilation, and drain-related barotrauma. No previous primary report of pneumomediastinum after autologous flap breast reconstruction was identified. **Conclusions**: Pneumothorax and pneumomediastinum are uncommon but potentially serious complications of ABR. They are rarely documented postoperatively; however, surgeons and anaesthetists must remain vigilant, particularly during exposure of the internal mammary vessels and airway management. Patient-specific assessment, symptom-directed imaging and multidisciplinary management may support early recognition to prevent morbidity and preserve the outcomes of reconstructive procedures.

## 1. Introduction

Breast reconstruction following mastectomy has undergone significant advancements since Czerny’s first recorded case in 1895. Early techniques throughout the 20th century primarily involved autologous reconstruction, utilising the patient’s own tissue, whereas implant-based methods have become increasingly prevalent since the early 21st century [1]. Although implant-based reconstruction is relatively popular, autologous breast reconstruction remains essential, particularly as an alternative in cases of implant failure or as the preferred option for patients undergoing radiotherapy. Abdominal free flaps, such as the deep inferior epigastric perforator (DIEP) flap, are favoured due to their superior aesthetic outcomes and muscle-sparing properties [2,3].

The population presenting for autologous reconstruction has changed over the past decade. Routine germline testing now identifies pathogenic variants in BRCA1, BRCA2 and other moderate-penetrance genes in younger women, many of whom elect bilateral risk-reducing mastectomy with immediate bilateral reconstruction [4]. Genomically driven surgical decision-making therefore increases the proportion of patients undergoing bilateral recipient-vessel dissection, doubling the anatomical exposure to the pleural reflection in a single operative episode. Precision oncology has thus reshaped the number of patients at risk of thoracic complications, even though the complications themselves remain rare. Understanding and communicating that risk at an individual level is a core objective of personalised reconstructive practice [5].

Pneumomediastinum is a rare condition characterised by the presence of gas in the mediastinum, while pneumothorax is characterised by gas in the pleural space. There are several theoretical mechanisms that can cause these complications postoperatively following ABR. This includes penetrating injury to the pleura and chest wall during internal mammary artery and vein dissection or periprocedural nerve block, or barotrauma during intubation and ventilation under general anaesthesia [6,7]. The dominant mechanism in non-traumatic pneumomediastinum is the Macklin effect, in which a transient rise in intra-alveolar pressure produces alveolar rupture and centripetal dissection of air along bronchovascular sheaths into the mediastinum [6]. Contemporary reviews emphasise that this process may occur without any recognised pleural breach and may be entirely radiologically occult on plain film [8,9].

The current literature regarding thoracic complications of autologous breast reconstruction is sparse. Discussion of the potential mechanisms of action, predisposing factors, assessment and management of these conditions is mainly limited to a few case series or case reports. No robust multivariate analyses are available to provide stronger evidence on this topic. Pneumothorax has been reported several times in retrospective studies, but often as an incidental statistic with no further insights offered, instead of the more common post-operative complications of breast reconstruction surgery.

As DIEP flap reconstruction and other autologous breast reconstructive surgeries are not commonly associated with pneumomediastinum or pneumothorax, there may be a case of under-reporting in the current literature. Complications of concern that are documented post-reconstructive surgery mostly include local complications of flap failure, seroma, wound dehiscence, wound necrosis and wound infections [7,10]. However, a survey of over 180 plastic surgeons in 2001 performed by Osborn and Stevenson revealed that one in three had experience with pneumothorax following breast augmentation surgery, suggesting that it may be a more common complication than it appears [11].

This literature review is centred on an institutional case of pneumomediastinum following bilateral DIEP flap reconstruction and examines the evidence for thoracic air complications after autologous breast reconstruction. We aim to synthesise current evidence, identify existing knowledge gaps, and provide insights relevant to clinical practice. The objectives are to quantify the incidence/frequencies of thoracic complications following breast reconstructive surgery and to inform both pre-operative counselling and post-operative monitoring. Additionally, the review will outline key considerations for the initial assessment and management of these complications.

## 2. Case Report

### 2.1. Patient Background and Genomic Risk Profile

A 41-year-old female with no significant past medical or surgical history presented following self-detection of a left breast mass. She was a non-smoker, not on regular medications, and had no prior abdominal or thoracic surgery. Imaging and core biopsy confirmed a 33 mm invasive carcinoma of the left breast, grade 3 (IC G3), with the following receptor profile: ER 3+ (90%), PR 3+ (50%), HER2-negative, Ki-67 75%. Germline testing confirmed a *BRCA1* pathogenic variant, informing the decision for bilateral risk-reducing mastectomy. Body mass index was 27.5 kg/m^2^. Preoperative haemoglobin was 104 g/L. She had no history of asthma, chronic obstructive pulmonary disease, connective tissue disorder, prior chest wall irradiation or prior thoracic intervention.

The patient completed neoadjuvant chemotherapy with paclitaxel and carboplatin prior to surgery. Sentinel lymph node biopsy of the left axilla (0 of 4 nodes positive) confirmed node-negative disease. Multidisciplinary team consensus supported proceeding to bilateral skin-sparing, nipple-sacrificing mastectomy with immediate bilateral DIEP flap reconstruction. It is relevant to the present discussion that the contralateral (right) mastectomy and reconstruction were performed for genomically defined risk reduction rather than for established malignancy; the bilateral operative burden—and hence bilateral recipient-vessel dissection—was a direct consequence of the germline result.

### 2.2. Operative Procedure

The patient underwent bilateral skin-sparing, nipple-sacrificing mastectomy followed by immediate bilateral DIEP flap breast reconstruction. The internal mammary vessels were utilised bilaterally as recipient vessels, consistent with standard practice. 15 fr Blake drains (gravity) were inserted bilaterally in the breast pocket.

Recipient vessel exposure was undertaken bilaterally at the third intercostal space using a rib-sacrificing with costal cartilage excision technique. A Gelpi retractor was used to maintain intercostal exposure. Both monopolar and bipolar diathermy was used for dissection and hemostasis. The parietal pleura was inspected directly at the conclusion of vessel preparation on both sides; no breach was identified. No intraoperative bubble test or sustained-inflation Valsalva manoeuvre was performed prior to closure. No regional anaesthesia or blocks were done pre/post operatively.

The anaesthetic record documented a Grade 1 direct laryngoscopy view, uncomplicated endotracheal intubation, and no intraoperative evidence of barotrauma. Ventilation was volume-controlled with a tidal volume of 6–8 mL/kg, positive end-expiratory pressure of 5 cmH_2_O, and peak airway pressures generally kept under 25–30 cmH_2_O. Total anaesthetic time was 8 h 17 min. No intraoperative complications were recorded by the operative or anaesthetic teams.

### 2.3. Perioperative Timeline

A structured timeline is provided (Table 1).

### 2.4. Post-Operative Course and Radiological Findings

In the immediate post-operative period, the patient developed hypotension and was found to be anaemic and was resuscitated with two units of packed red blood cells. Bilateral flap perfusion was confirmed satisfactory on evening flap assessment. Haemoglobin fell from pre-op 104 g/L to a nadir of 78 g/L, recovering to 92 g/L after transfusion. This episode responded promptly to blood product replacement and is best explained by perioperative blood loss; it is temporally distinct from, and preceded, the radiological diagnosis of pneumomediastinum.

During the overnight period, the patient experienced recurrent haemodynamic instability with systolic blood pressure as low as 92 mmHg and a heart rate of 120 beats per minute. Oxygen saturation was 97% on room air. Electrocardiography demonstrated sinus tachycardia. Abdominal examination was soft and non-tender; the wound dressings were clean with no evidence of strike-through or overt haemorrhage. Fluid resuscitation was initiated.

On the morning of post-operative day one, a Medical Emergency Team (MET) activation was triggered for tachycardia (146 beats per minute). At this instance, the patient was normotensive. Oxygen saturation was 95% on room air. Chest auscultation was clear bilaterally, and ECG confirmed sinus tachycardia without ischaemic changes. The patient reported pleuritic chest pain on movement and nausea; she was receiving analgesia via patient-controlled analgesia (PCA), which was being actively titrated by the acute pain service. Notably, she did not report dyspnoea at rest, and there was no subcutaneous emphysema, no Hamman’s sign and no voice change on repeated examination.

**Figure 1:**
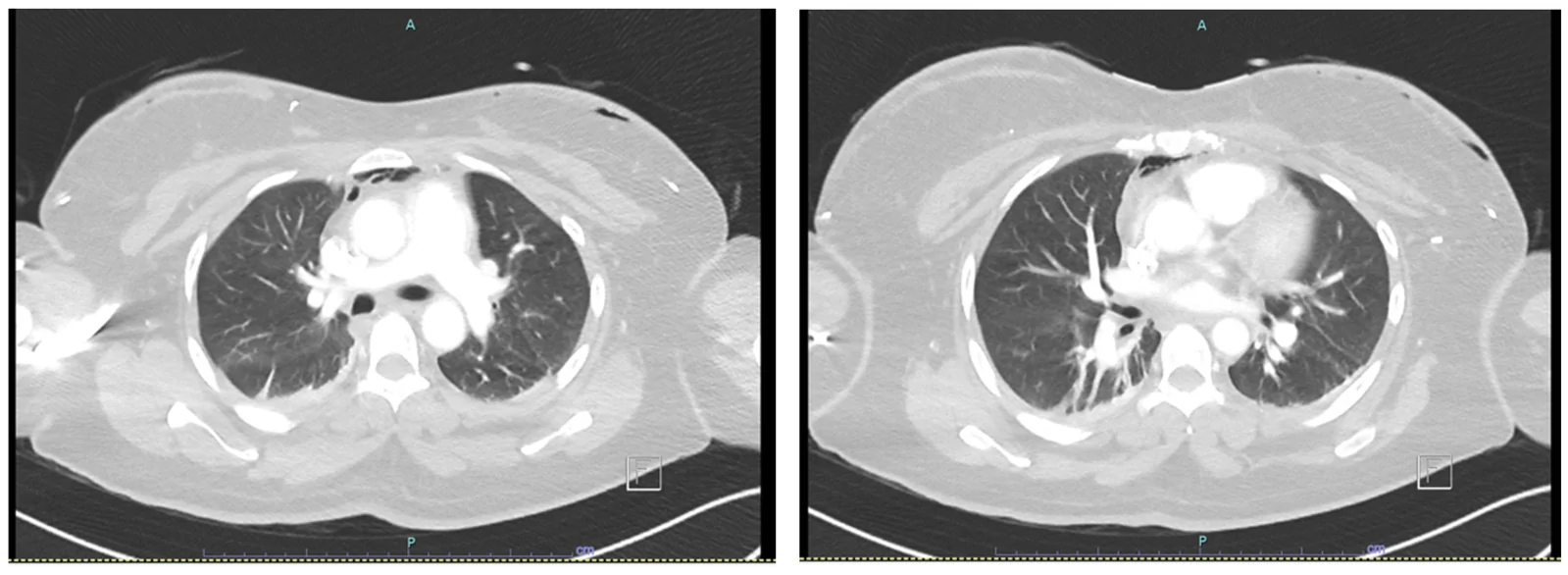
Initial CTPA to rule out Pulmonary embolism showing Pneumomediastinum.

Given the persistence of tachycardia, pleuritic chest pain, and low systolic blood pressure, a decision was made to proceed to CT pulmonary angiography (CTPA) to exclude pulmonary embolism and occult haemorrhage. CTPA demonstrated moderate-volume pneumomediastinum with no evidence of pulmonary embolism (Figure 1). No pneumothorax was identified. No anatomical variations, pectus deformities, or pre-existing structural thoracic abnormalities were confirmed by radiology review.

Radiological description (Figure 1). On axial CTPA images locules of extraluminal gas were demonstrated within the anterior mediastinum. The visceral and parietal pleural surfaces were apposed bilaterally with no pleural gas. Lung parenchyma demonstrated subsegmental collapse on the lower lobes bilaterally, with no bullae, blebs, contusion or interstitial emphysema. The tracheobronchial tree was intact to the segmental level. There was no pneumopericardium and no cervical or chest-wall subcutaneous emphysema. Estimated volume of mediastinal gas was reported as moderate-volume by the reporting radiologist’s assessment.

### 2.5. Multidisciplinary Management and Outcome

**Figure 2:**
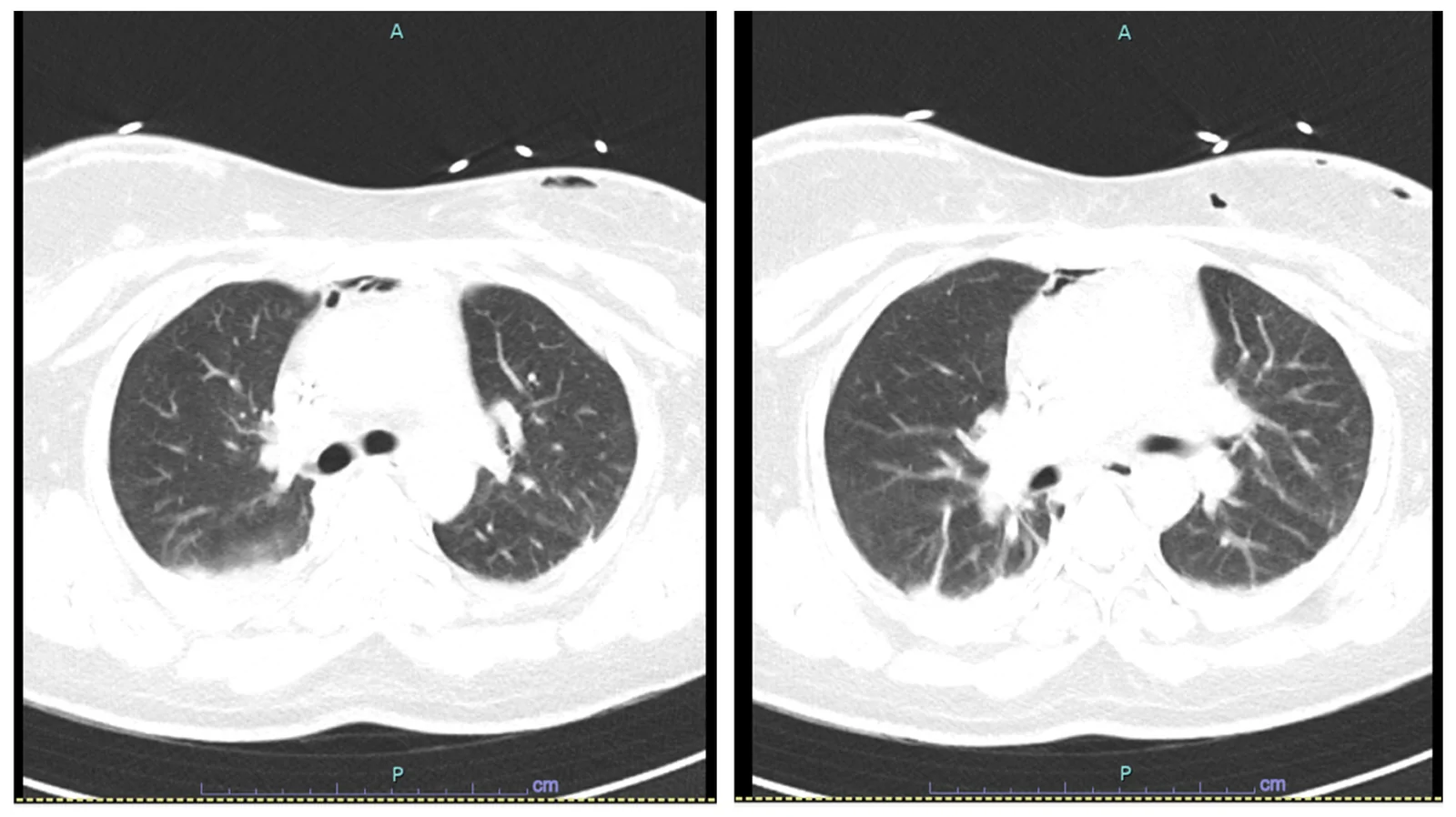
CT chest showing stable pneumomediastinum.

The CTPA findings were discussed with the cardiothoracic surgical team, who advised against immediate surgical intervention and recommended admission to the high-dependency unit (HDU) for close haemodynamic monitoring. Given the clinical concern regarding a secondary cause of pneumomediastinum, a CT chest with oral contrast was subsequently performed to exclude oesophageal perforation. This demonstrated stable pneumomediastinum with an intact oesophagus and no evidence of perforation or mediastinal fluid (Figure 2).

Radiological description (Figure 2). Delayed CT of the chest with oral contrast, performed 21 h after the index CTPA, demonstrated an unchanged distribution and volume of anterior mediastinal gas relative to the prior study. The oesophageal wall was of normal thickness throughout its cervical, thoracic and abdominal segments, with no mural discontinuity and no extraluminal contrast. There was no mediastinal fluid collection, no fat stranding to suggest mediastinitis, and no pleural effusion. The absence of mediastinal fluid is of particular diagnostic value, as its presence is a recognised discriminator favouring oesophageal perforation over a primary alveolar mechanism.

Following exclusion of oesophageal injury, the patient was stepped down from HDU to a surgical ward for ongoing conservative management. This comprised analgesia, supplemental oxygen, close nursing observation of haemodynamic parameters and oxygen saturation, and serial clinical review. Flap viability was preserved throughout.

The patient’s haemodynamics and oxygen saturations remained stable over the subsequent days. She was discharged home on post-operative day seven, without the requirement for thoracic intervention. At outpatient review, she was asymptomatic and thus no further imaging to confirm resolution was done.

### 2.6. Clinical Interpretation and Causal Attribution

The relationship between the pneumomediastinum and the patient’s postoperative haemodynamic disturbance cannot be established with certainty. The initial hypotension occurred within hours of a prolonged bilateral procedure and improved following fluid resuscitation and transfusion of two units of packed red blood cells. Perioperative blood loss, postoperative anaemia, intravascular volume depletion, fluid shifts, pain, nausea, and the physiological stress of major surgery may therefore have contributed to the early haemodynamic changes. However, the available clinical information does not permit attribution to a single cause.

Persistent sinus tachycardia subsequently developed in association with pleuritic chest pain. At that time, blood pressure was preserved, oxygen saturation remained near normal on room air, and chest examination was unremarkable. CT demonstrated pneumomediastinum without pneumothorax, oesophageal perforation, or features of tension physiology. These findings make it unlikely that the pneumomediastinum alone accounted for the patient’s haemodynamic presentation. Nevertheless, mediastinal air may have contributed to the pleuritic discomfort and associated sympathetic tachycardia. The overall clinical course is therefore most appropriately interpreted as multifactorial, with possible contributions from perioperative blood loss, volume status, pain, and pneumomediastinum.

The case remains clinically instructive because the pneumomediastinum was not apparent on physical examination and was detected only when cross-sectional imaging was performed to investigate persistent tachycardia and chest pain. Once identified, the finding required exclusion of clinically important secondary causes, particularly oesophageal or tracheobronchial injury. In this patient, CT with oral contrast demonstrated no oesophageal perforation or mediastinal fluid collection, and cardiothoracic review supported conservative management. The patient remained stable and did not require ventilatory support, decompression, or operative intervention.

This presentation also illustrates a potential mechanism of under-ascertainment. Small or moderate postoperative mediastinal air collections may remain undetected when symptoms are mild or non-specific and routine cross-sectional imaging is not performed. However, the available literature does not establish the frequency of asymptomatic or minimally symptomatic pneumomediastinum following autologous breast reconstruction, and under-reporting should therefore be considered a possibility rather than a confirmed phenomenon.

The mechanism of pneumomediastinum in this case remains uncertain. Oesophageal perforation, tracheobronchial injury, pneumothorax, and major pulmonary parenchymal disease were not identified radiologically. No intraoperative pleural breach was documented. Possible explanations include a Macklin-type alveolar air-leak mechanism associated with positive-pressure ventilation or emergence from anaesthesia, or tracking of air through postoperative tissue planes or drain pathways. The confinement of gas to the mediastinum without extensive cervical or chest-wall subcutaneous emphysema may be compatible with an alveolar mechanism, but this distribution is not diagnostic. Accordingly, these explanations should be regarded as plausible hypotheses rather than demonstrated causes. Although a causal relationship between pneumomediastinum and the patient’s symptoms is not proven, its detection in temporal association with persistent tachycardia and pleuritic chest pain supports its consideration within the differential diagnosis of otherwise unexplained postoperative cardiopulmonary symptoms.

## 3. Methods

We conducted a focused search to synthesise evidence from the published literature for this literature review, capturing all relevant reports describing thoracic complications associated with ABR. The population of interest comprised adult patients undergoing autologous or free-flap breast reconstruction, including DIEP, TRAM, and latissimus dorsi flaps, following mastectomy. The exposure of interest included surgical or anaesthetic factors that may be associated with thoracic injury. The outcomes of interest were the development, mechanism, clinical presentation, and management of pneumothorax or pneumomediastinum (Figure S1).

A comprehensive search was conducted across PubMed, MEDLINE- Ovid, and Wiley Online Library, supplemented by searches in the Journal of Surgical Case Reports and BMJ Case Reports to capture individual case reports not indexed in major databases. The search was completed on 3 June 2026, with only English language restrictions applied. Search terms combined relevant keywords and MeSH terms available in Supplemental Table S2. All retrieved studies were imported into Microsoft Excel software for screening and duplicate removal.

Two authors independently reviewed titles and abstracts, and full-text assessment was conducted using predefined inclusion and exclusion criteria. Studies were included if they reported pneumothorax or pneumomediastinum as a perioperative or postoperative complication of autologous breast reconstruction, regardless of study design, provided they contained original patient data and sufficient detail on presentation, mechanism, or management. Studies were excluded if they described thoracic complications unrelated to autologous reconstruction, animal experiments, or conference abstracts without accessible data, or if full texts could not be obtained or translated.

Two independent reviewers manually extracted data using a standardised form to analyse key variables, including breast reconstruction technique, perioperative airway and ventilation, type and timing of complication, symptoms, proposed mechanism of injury and management. Additionally, patient risk factors like prior thoracic surgery, modifiable and unmodifiable factors, and follow-up duration were examined.

A descriptive synthesis of study-specific reported frequencies of thoracic complications was conducted across the selected studies. The analysis also identified proposed mechanisms of injury during preoperative and intraoperative periods. Additionally, risk factors for developing thoracic complications were noted, and the management strategies implemented post-thoracic injury.

The quality of the five included studies was evaluated using the Joanna Briggs Institute (JBI) Critical Appraisal Checklists, with appropriate tools selected for each study design. The overall quality of evidence on this topic is low, primarily comprising Level IV evidence (case reports and series) and Level III/IV evidence (retrospective reviews). The case reports were comprehensive, providing clear descriptions of clinical presentation, management, and proposed mechanisms. Retrospective studies, which serve as the sole source of incidence data, exhibit a moderate-to-high risk of bias due to their design, including information bias, potential under-reporting, and limited control for confounding variables.

## 4. Results

Five primary studies met the revised eligibility criteria and reported pneumothorax following autologous flap breast reconstruction (Table 2). These comprised three retrospective series or cohort studies and two case reports. The procedures evaluated included free-flap reconstruction using a rib-sparing intercostal-space approach to the internal mammary vessels, extended latissimus dorsi flap reconstruction, DIEP flap reconstruction using internal mammary recipient vessels, and muscle-sparing TRAM flap reconstruction [12,13,14,15,16].

Three studies provided defined procedural denominators: 463 free-flap reconstructions using rib-sparing internal mammary vessel exposure, 749 extended latissimus dorsi reconstructions, and 180 patients undergoing DIEP reconstruction involving 283 internal mammary vessel dissections [12,13,14]. The remaining two publications were individual case reports and therefore did not permit estimation of event frequency [15,16] (Table 2).

No published primary report of pneumomediastinum following autologous flap breast reconstruction was identified. The pneumomediastinum described in the present institutional case was therefore considered separately from the published pneumothorax evidence and was not included in any study-level frequency calculation.

Darcy et al. reported one pneumothorax among 463 microvascular breast reconstructions using a rib-sparing intercostal-space approach to the internal mammary vessels, corresponding to a study-specific frequency of approximately 0.22% [12]. Gandamihardja et al. identified three pneumothoraces among 749 extended latissimus dorsi flap reconstructions (0.4%), comprising two simple pneumothoraces and one tension pneumothorax [13]. Kelling et al. reported four pneumothoraces among 180 patients undergoing DIEP flap reconstruction, equivalent to 2.2% per patient and 1.4 events per 100 internal mammary vessel dissections [14].

These estimates were not pooled because the studies differed in reconstructive technique, denominator, outcome ascertainment, perioperative surveillance, and reporting practices. The higher study-specific frequency reported by Kelling et al. should therefore not be interpreted as evidence that DIEP reconstruction carries a greater risk than other autologous techniques. Differences in surveillance and case detection may have influenced the reported frequencies, and none of the studies was designed to compare risk between reconstructive procedures.

Neither the retrospective studies nor the case reports provided a reliable population-level estimate of symptomatic pleural injury following autologous breast reconstruction.

Most events occurred intraoperatively or during the immediate postoperative period, although one pneumothorax presented on postoperative day four [13]. Presentation ranged from asymptomatic radiographic findings to dyspnoea, hypoxaemia, tachycardia, increased airway pressures, hypotension, and tension physiology. Reekie et al. described flap venous congestion as the first sign of an intraoperative tension pneumothorax [15]. Diagnosis was based on intraoperative recognition, chest radiography, CT, or targeted assessment for pleural leakage.

Direct pleural injury was confirmed in only one event, involving electrocautery during internal mammary vessel exposure [14]. Other proposed mechanisms—including retractor injury, pleural breach during costal cartilage excision, and injury during latissimus dorsi elevation—were inferred from the operative context rather than directly observed [13,14,15,16]. Gelpi retractor use, bilateral reconstruction, radiotherapy, and anatomical factors were reported descriptively but could not be considered established risk factors because of the small number of events and absence of adjusted analyses.

Management reflected clinical severity (Table 3). Small stable pneumothoraces were observed with supplemental oxygen, while clinically significant events required intercostal or pigtail drainage. Tension pneumothorax required immediate needle decompression followed by chest drainage [13,15]. Reported cases generally resolved without persistent respiratory impairment or flap loss attributable to the thoracic complication.

JBI appraisal identified moderate methodological concerns in the retrospective studies and inherently very low certainty in the case reports presented in Supplemental Table S1. Consequently, the evidence supports recognition of rare possible complications and their management, but does not permit pooled incidence estimates, procedural comparisons, or reliable risk-factor conclusions.

## 5. Discussion

This review identified a small and heterogeneous body of evidence describing pneumothorax after autologous flap breast reconstruction. Across the five included primary studies, events were uncommon, but differences in reconstructive technique, denominator, surveillance, and outcome ascertainment prevented calculation of a pooled incidence or comparison between procedures. No previous primary report of pneumomediastinum following autologous breast reconstruction was identified. The present case therefore contributes by describing the investigation and conservative management of pneumomediastinum following bilateral DIEP reconstruction rather than by establishing its frequency or cause.

The study-specific pneumothorax frequencies of approximately 0.22%, 0.4%, and 2.2% were derived from different operative populations and should be interpreted independently [12,13,14]. Darcy et al. evaluated a rib-sparing intercostal-space approach to the internal mammary vessels, Gandamihardja et al. examined extended latissimus dorsi reconstruction, and Kelling et al. reviewed DIEP reconstruction using internal mammary recipient vessels [12,13,14]. Differences in surveillance may also have influenced event detection, as Kelling et al. incorporated intraoperative assessment and early postoperative imaging, which may have identified small or minimally symptomatic pneumothoraces [14]. The higher frequency in that study therefore does not demonstrate that DIEP reconstruction carries greater risk than other autologous techniques.

The evidence also does not establish that every reported pneumothorax was directly caused by the reconstructive procedure. A technical pleural injury was directly observed in only one event reported by Kelling et al.; in the remaining events, mechanisms were inferred from operative timing, anatomy, or physiological response [13,14,15,16]. Symptomatic pleural injury attributable to autologous breast reconstruction appears uncommon, but the available studies are insufficient to determine its true frequency.

Several procedural and patient characteristics were described among affected patients, but the low number of events prevents reliable risk estimation. In the Kelling series, a Gelpi retractor was used in all four pneumothorax cases, and three events occurred following bilateral reconstruction; however, no adjusted analysis was possible [14]. These observations are best regarded as hypothesis-generating and should not be used to guide definitive risk stratification.

The most frequently proposed mechanism is inadvertent injury to the parietal pleura during recipient-vessel exposure or flap elevation. Internal mammary vessel dissection occurs close to the pleura, particularly where the intercostal space is narrow or costal cartilage is removed. Kelling et al. documented one electrocautery-related pleural injury and suspected that deep retraction may have contributed to other events [14]. Patel and Malata attributed a postoperative pneumothorax to internal mammary vessel preparation and third costal cartilage excision, although the pleural defect was not directly visualised [16]. In extended latissimus dorsi reconstruction, Gandamihardja et al. and Reekie et al. proposed that an unrecognised pleural breach occurred during flap elevation, with positive-pressure ventilation contributing to enlargement or tension physiology [13,15].

Implant and tissue-expander reconstruction were excluded from the primary synthesis; these reports provide relevant anatomical context. The pleura may be particularly vulnerable where anterior chest-wall tissues are thin, scarred, or less compliant, because dissection and electrocautery occur close to the intercostal musculature and parietal pleura. Reports of pleural entry during submuscular pocket formation in patients with thin intercostal tissues, and during dissection through previously irradiated and scarred tissue, support the biological plausibility that reduced tissue thickness or fibrosis may make chest-wall dissection more technically demanding [17,18]. These findings cannot be directly extrapolated to free-flap reconstruction, but they provide a reasonable anatomical basis for considering body habitus, prior radiation, and previous chest-wall surgery during operative planning.

A mechanistically distinct pathway is the Macklin effect, in which alveolar rupture permits air to track along the bronchovascular sheaths into the mediastinum without pleural disruption [6]. Positive-pressure ventilation, coughing, or transient increases in intra-alveolar pressure may contribute, although excessive airway pressures do not need to be documented for the mechanism to remain possible. Air may also track through surgically altered tissue planes or along drain pathways, as proposed in a non-autologous breast surgery case involving pneumomediastinum and subcutaneous emphysema demonstrating another probable mechanism [19].

The cause of pneumomediastinum in the present patient remains uncertain. No pneumothorax, oesophageal perforation, tracheobronchial injury, pulmonary embolism, or recognised intraoperative pleural defect was identified. The anaesthetic record documented uncomplicated intubation and no recognised barotrauma. The relationship between the pneumomediastinum and the postoperative haemodynamic disturbance also cannot be established with certainty. Hypotension developed shortly after a prolonged bilateral procedure and improved following fluid resuscitation and transfusion of two units of packed red blood cells. Perioperative blood loss, postoperative anaemia, intravascular volume depletion, fluid shifts, pain, nausea, and the physiological stress of major surgery may therefore have contributed. Persistent sinus tachycardia subsequently occurred with pleuritic chest pain, prompting CTPA. Pneumomediastinum may have contributed to the patient’s discomfort and sympathetic tachycardia; however, preserved oxygenation, absence of pneumothorax or tension physiology, and the lack of thoracic intervention make it unlikely to have been the sole explanation. The clinical course is therefore most appropriately interpreted as multifactorial. Direct pleural injury is not supported by the absence of pleural gas or a recognised intraoperative breach, although a small occult injury cannot be completely excluded. A Macklin-type alveolar mechanism related to positive-pressure ventilation or emergence from anaesthesia remains plausible. Tracking of air through surgical or drain-related planes is another possibility; however, the absence of cervical or chest-wall subcutaneous emphysema makes a superficial tracking pathway less evident. None of these mechanisms can be confirmed from the available findings.

The case remains clinically informative because pneumomediastinum was identified during post operative investigation of tachycardia and pleuritic chest pain. Although rare, the case signifies thoracic air complication as an important differential when prolonged hemodynamic instability persists post operatively. This case therefore demonstrates the importance of distinguishing an incidental or contributory radiological finding from a haemodynamically significant thoracic complication and supports symptom-directed investigation rather than routine postoperative thoracic imaging.

The relevance to personalised medicine lies in tailoring surgical planning, surveillance, and escalation to the individual patient rather than applying uniform investigation or intervention. Relevant considerations may include chest-wall anatomy, previous thoracic surgery or radiotherapy, body habitus, unilateral or bilateral reconstruction, recipient-vessel choice, and the anticipated method of internal mammary exposure. In the present case, germline BRCA1 status influenced the decision to undertake bilateral mastectomy and bilateral immediate reconstruction. This increased the extent of recipient-vessel dissection during a single operation, although bilateral reconstruction has not been established as an independent risk factor for thoracic air complications. The case nevertheless illustrates how genomic information can influence both the oncological decision and the anatomical exposure associated with reconstructive care. Existing staging imaging may also provide information regarding chest-wall anatomy, intercostal-space width, previous surgical change, or pectus deformity. Such information may assist operative planning in selected patients, although there is currently no evidence that routine preoperative measurements predict pneumothorax.

Meticulous dissection is important during internal mammary vessel exposure and latissimus dorsi elevation. When pleural injury is suspected, direct inspection, a Valsalva manoeuvre, or a saline bubble test may assist recognition, although routine testing is not supported [20,21]. Recognised defects may be repaired by direct suture, fat-plug closure, or fascial or muscle coverage [22,23]. Postoperative investigation should be symptom-directed, with chest radiography, point-of-care ultrasound, or CT selected according to clinical severity and diagnostic uncertainty. Small stable pneumothoraces may be observed, whereas clinically significant or tension pneumothorax requires drainage or immediate decompression. Chest-drain placement should be coordinated with the reconstructive team to avoid the internal mammary pedicle [16]. Stable isolated pneumomediastinum may be managed conservatively once major secondary causes are excluded; antibiotics are not routinely required without suspected infection [24].

This review is limited by the small number of eligible studies, retrospective ascertainment, inconsistent surveillance, heterogeneous procedures, and very low event counts. Case reports provide useful information about recognition and management but cannot estimate incidence or identify risk factors. The retrospective studies provide procedural denominators but differ in reconstructive technique and outcome detection; no pooled analysis was therefore undertaken.

## 6. Conclusions

Thoracic air complications following autologous breast reconstruction are uncommon, but the available evidence is sparse, heterogeneous, and largely derived from retrospective series and case reports. The current literature does not support a pooled incidence estimate, comparison between reconstructive techniques, or identification of validated patient- or procedure-specific risk factors. In the present case, pneumomediastinum following bilateral DIEP flap reconstruction was identified during investigation of postoperative tachycardia and pleuritic chest pain. No definitive mechanism was established, and the haemodynamic disturbance was considered multifactorial rather than attributable to pneumomediastinum alone.

A personalised approach to these complications should integrate individual chest-wall anatomy, previous surgery or radiotherapy, reconstructive technique, recipient-vessel exposure, anaesthetic factors, and the postoperative clinical trajectory. Investigation should be symptom-directed, with escalation based on physiological severity and competing diagnoses rather than routine imaging. Where pneumothorax or pneumomediastinum is identified, management should be tailored to clinical stability and the presence of associated pleural injury. Further prospective, denominator-defined studies are required to better characterise frequency, mechanisms, and clinically relevant risk factors.

## Figures and Tables

**Table 1:** Perioperative timeline of the illustrative case. SSM, skin-sparing mastectomy; IMV, internal mammary vessels; SBP, systolic blood pressure; MET, medical emergency team; PCA, patient-controlled analgesia; CTPA, computed tomography pulmonary angiography; HDU, high-dependency unit.

Time Point	Clinical Event	Interpretation/Action
Day 0 (intra-op)	Bilateral SSM with immediate bilateral DIEP; IMV recipients bilaterally. No recognised pleural breach; Grade 1 intubation; no documented barotrauma.	Uneventful by intraoperative record.
Day 0, early post-op	Hypotension 92/65 mmHg and tachycardia 120 bpm. Transfused 2 units packed red blood cells due to Hb of 78. Flap perfusion satisfactory on evening check.	Attributed to perioperative blood loss and anaesthetic vasodilatation. Responded to transfusion.
Day 0, overnight	Abdomen soft, dressings dry, no strike-through. Fluid resuscitation given. Adequate resuscitation with post operative transfusion and intravenous fluids.	Ongoing assessment for occult haemorrhage. No respiratory signs at this stage.
Day 1, morning	MET call for HR 146 bpm; normotensive at this point. SpO_2_ 95% room air. Chest clear on auscultation. Sinus tachycardia, no ischaemic change.	Isolated tachycardia without hypotension—differential broadened to include pulmonary embolism.
Day 1, daytime	Remains tachycardic with pleuritic chest pain on movement and nausea. PCA in situ, titrated by acute pain service. Lowest recorded SBP 87 mmHg	Symptoms non-specific; consistent with postoperative pain and opioid effect.
Day 1	CTPA: moderate volume pneumomediastinum. No pulmonary embolus. No pneumothorax. No anatomical variant or pectus deformity on radiologist review.	Unanticipated finding. Cardiothoracic surgery consulted; HDU admission for observation; no intervention advised.
Day 2	Decreasing drain tube outputs and CT chest with oral contrast as requested by cardiothoracic: stable pneumomediastinum, intact oesophagus, no mediastinal fluid or contrast extravasation.	Oesophageal perforation excluded—the principal reason for escalating imaging.
Day 3–6	Stepped down from HDU. Analgesia, supplemental oxygen, serial observation. Flaps viable throughout. Remained normotensive and resolved tachycardia. Drains removed prior to discharge	Conservative management.
Day 7	Discharged home. Observations and saturations stable. No thoracic intervention required.	Clinical resolution.
Follow-up	Seen 1 week after discharge. Ongoing abdominal pain, no shortness of breath of pre-syncope/syncope.	Subsequent follow up in clinic with improving pain. However, developed abdominal wound dehiscence requiring debridement.

**Table 2:** Primary studies reporting pneumothorax after autologous flap breast reconstruction. Frequencies are study-specific and were not pooled. IMV, internal mammary vessels; PTX, pneumothorax; POD, postoperative day.

Study	Design	Autologous Procedure	Denominator	Thoracic event	Study-Specific Frequency	Timing
Darcy et al. [12] (2011)	Retrospective technical series	Free-flap reconstruction; rib-sparing intercostal-space IMV exposure	463 reconstructions	1 pneumothorax	1/463 (0.22%)	Not reported in accessible summary
Gandamihardja et al. [13] (2013)	Retrospective series	Extended latissimus dorsi flap	749 reconstructions	2 simple PTX; 1 tension PTX	3/749 (0.4%)	Two immediate; one POD4
Kelling et al. [14] (2021)	Single-centre retrospective cohort	DIEP flap using internal mammary recipient vessels	180 patients; 283 IMV dissections	4 pneumothoraces	2.2% per patient; 1.4/100 dissections	Intraoperative or immediate postoperative
Reekie et al. [15] (2014)	Case report	Extended latissimus dorsi flap	1 patient	Intraoperative tension PTX	Not applicable	Near completion of surgery
Patel and Malata [16] (2010)	Case report	Muscle-sparing TRAM flap using IMVs	1 patient	Large postoperative PTX	Not applicable	Immediate postoperative period

**Table 3:** Clinical presentation, proposed mechanisms and management. Proposed mechanisms are distinguished from confirmed intraoperative findings.

Study	Presentation/Diagnosis	Proposed Mechanism	Management/Outcome	Certainty of Mechanism
Darcy et al. [12]	Details not reported in accessible summary	Pleural injury during recipient-vessel exposure was possible	Not reported	Unclear
Gandamihardja et al. [13]	Respiratory symptoms and reduced oxygen saturation; one tension event	Possible pleural breach during latissimus dorsi elevation	Chest drainage; immediate needle decompression for tension PTX	Proposed; no defect confirmed in extracted data
Kelling et al. [14]	Asymptomatic to tachycardia, tachypnoea and increased oxygen requirement; intraoperative test/CXR	One confirmed electrocautery pleural injury; retractor injury suspected in others	Observation/O_2_ for small stable events; pigtail drainage for clinically significant PTX; direct repair with fat plug in recognised tear	Confirmed in one event; inferred in remaining events
Reekie et al. [15]	Flap venous congestion followed by hypotension, tachycardia, hypoxia and increased airway pressures	Pleural injury during flap elevation with progression under positive-pressure ventilation	Needle decompression followed by chest drain; flap congestion resolved	Physiologically plausible but not directly visualised
Patel and Malata [16]	Immediate dyspnoea and desaturation; CXR/CT	Pleural breach during IMV preparation and third-costal cartilage resection	Intercostal drainage; full recovery without flap compromise	Strong temporal association; defect not directly documented

## Data Availability

No new data were created or analyzed in this study. Data sharing is not applicable to this article.

## Supplementary Materials

The following supporting information can be downloaded at: https://www.mdpi.com/article/10.3390/jpm16090458/s1, Figure S1: PRISMA flow diagram for included studies; Table S1: JBI critical appraisal. Y, yes; N, no; U, unclear; NA, not applicable. Overall judgements were made by consensus without numerical cut-offs; Table S2: Search Strings.
