# Supplementary material for: Optimising Autologous Breast Reconstruction: Pneumothorax and Pneumomediastinum—A Literature Review and First Reported Case of Pneumomediastinum Following Bilateral DIEP Flap Reconstruction
Source: J Pers Med. 2026 Aug 30;16(9):458. doi: 10.3390/jpm16090458 (PMC13608144; doi:10.3390/jpm16090458)
Supplement: Supplementary file 1 [file jpm-16-00458-s001.zip › jpm-4420117-supplementary.pdf]

Supplemental data

This study was designed as a focused literature review accompanying an illustrative case report rather than as a systematic review or meta-analysis. A review protocol was not prospectively registered, and PRISMA 2020 was not prespecified as a reporting framework. The review nevertheless used predefined eligibility criteria, independent screening by two authors, structured data extraction, and study-design–appropriate Joanna Briggs Institute (JBI) critical appraisal (Table S1). Given the rarity of the outcomes, heterogeneity of study designs and reconstructive techniques, and predominantly descriptive nature of the available evidence, the synthesis was narrative and no pooled incidence estimate or meta-analysis was planned.

Search Strategy and Eligibility

A focused structured literature search was undertaken to identify primary reports of pneumothorax or pneumomediastinum occurring during or after autologous breast reconstruction. The population of interest comprised adults undergoing autologous flap breast reconstruction, including deep inferior epigastric perforator (DIEP), transverse rectus abdominis myocutaneous (TRAM), muscle-sparing TRAM, superficial inferior epigastric artery (SIEA), and latissimus dorsi flap reconstruction following mastectomy.

Electronic searches were performed in PubMed and MEDLINE Ovid. Supplementary searches were undertaken through Wiley Online Library and targeted searches of the Journal of Surgical Case Reports and BMJ Case Reports. The final search was performed on 03/06/2026. No date restriction was applied, and only English-language articles were included / No date or language restrictions were applied.

The search combined terms relating to autologous breast reconstruction with terms describing thoracic air complications. Controlled vocabulary was combined with free-text terms, including “breast reconstruction”, “autologous breast reconstruction”, “free flap”, “DIEP”, “TRAM”, “latissimus dorsi”, “pneumothorax”, “pneumomediastinum”, “pleural injury”, “pleural breach”, “thoracic complication”, and “barotrauma”. The complete search strategies are provided in Supplementary Table S2.

Studies were eligible if they contained original patient data describing pneumothorax or pneumomediastinum as an intraoperative, perioperative, or postoperative complication of autologous flap breast reconstruction. Case reports, case series, and observational cohort or retrospective studies were eligible. Studies involving implant-only reconstruction, reduction mammoplasty, breast-conserving surgery without autologous flap reconstruction, or other non-autologous breast procedures were excluded from the primary synthesis. Narrative reviews, conference abstracts without sufficient patient-level data, animal studies, and reports for which adequate full-text information could not be obtained were also excluded. Non-autologous reports relevant to mechanisms, diagnosis, or management were considered only as contextual literature in the Discussion and did not contribute to study counts or frequency estimates.

Two authors independently screened titles and abstracts and subsequently assessed potentially eligible full-text articles. Disagreements were resolved by. Data were extracted using a standardised form capturing reconstructive technique, denominator, timing and type of thoracic complication,

clinical presentation, proposed mechanism, diagnostic assessment, management, follow-up, and reported patient- or procedure-related factors.

Figure S1: PRISMA flow diagram for included studies

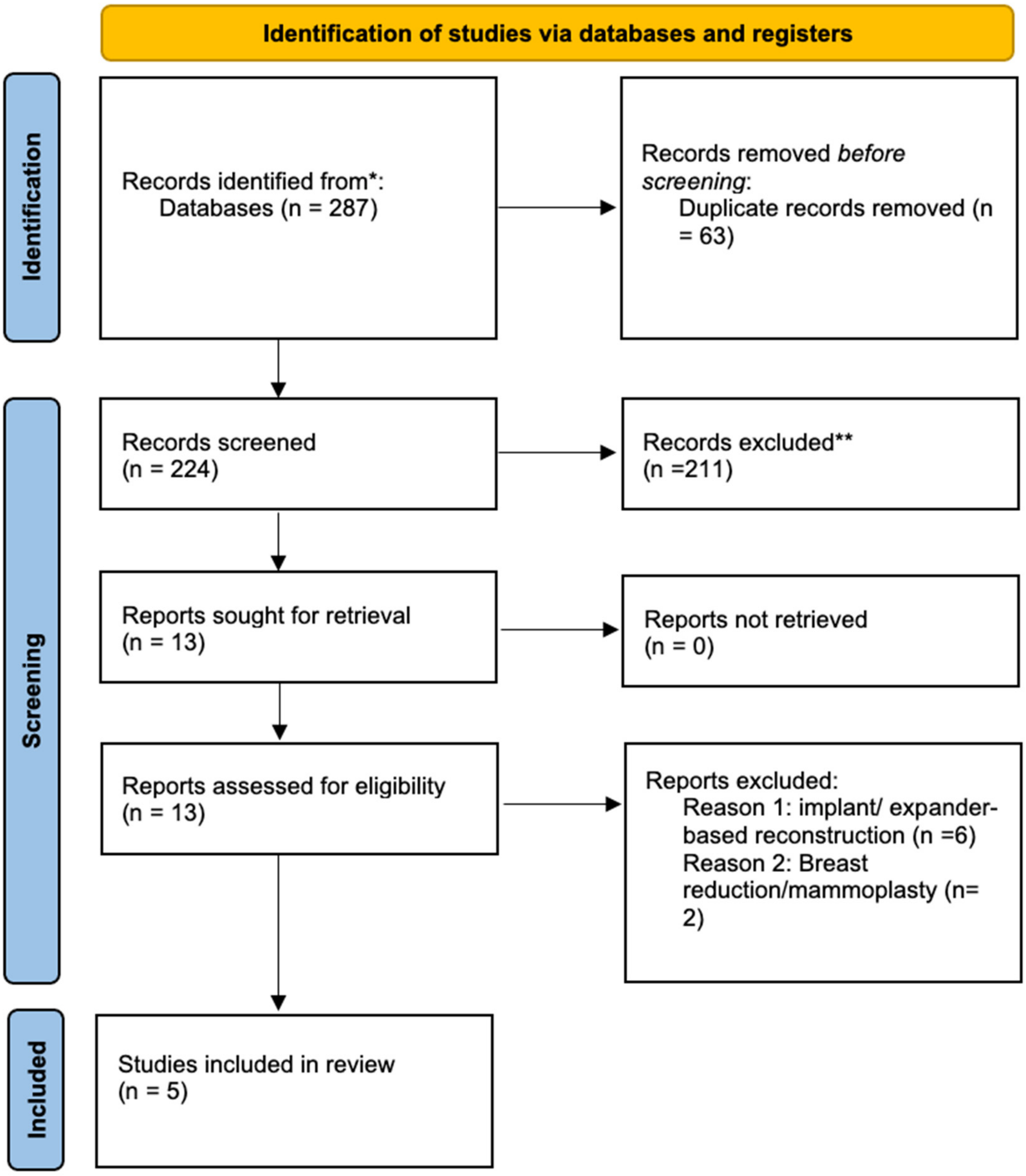

Table S1. JBI critical appraisal. Y, yes; N, no; U, unclear; NA, not applicable. Overall judgements were made by consensus without numerical cut-offs.

Study / JBI tool	Item-level appraisal	Overall methodological concern	Influence on synthesis
Darcy et al. [12]	Q1 U; Q2 Y; Q3 Y; Q4 U; Q5 U; Q6 U; Q7 Y; Q8 Y; Q9 Y; Q10 Y	Moderate concerns	Useful denominator; limited patient-level and event detail
Gandamihardja et al. [13]	Q1 U; Q2 Y; Q3 Y; Q4 U; Q5 U; Q6 Y; Q7 Y; Q8 U; Q9 Y; Q10 Y	Moderate concerns	Useful denominator; possible incomplete event capture and limited follow-up
Kelling et al. [14]	Q1 NA; Q2 Y; Q3 Y; Q4 Y; Q5 N; Q6 Y; Q7 Y; Q8 Y; Q9 U; Q10 N; Q11 Y	Moderate concerns	Best ABR-specific incidence data; low event count and no adjusted analysis
Reekie et al. [15]	Q1 Y; Q2 Y; Q3 Y; Q4 Y; Q5 Y; Q6 Y; Q7 Y; Q8 Y	Low reporting concerns; inherently very low certainty	Supports clinical recognition and management, not incidence or risk estimation
Patel and Malata [16]	Q1 Y; Q2 Y; Q3 Y; Q4 Y; Q5 Y; Q6 Y; Q7 Y; Q8 Y	Low reporting concerns; inherently very low certainty	Supports mechanism and management discussion, not incidence or risk estimation

Table S2: Search Strings

Database	Strings
Pubmed	("Mammaplasty"[Mesh] OR "Breast Implantation"[Mesh] OR "Surgical Flaps"[Mesh] OR "Perforator Flap"[Mesh] OR "Myocutaneous Flap"[Mesh] OR "Free Tissue Flaps"[Mesh] OR "Mastectomy"[Mesh] OR "breast reconstruction"[tiab] OR "autologous breast reconstruction"[tiab] OR "DIEP"[tiab] OR "deep inferior epigastric perforator"[tiab] OR "TRAM"[tiab] OR "transverse rectus abdominis"[tiab] OR "SIEA"[tiab] OR "superficial inferior epigastric"[tiab] OR "latissimus dorsi"[tiab] OR "free flap"[tiab] OR "reduction mammoplasty"[tiab] OR "tissue expander"[tiab])<br><br>("Pneumothorax"[Mesh] OR "Mediastinal Emphysema"[Mesh] OR "Subcutaneous Emphysema"[Mesh] OR "Barotrauma"[Mesh] OR "Pneumoperitoneum"[Mesh] OR pneumothorax[tiab] OR pneumomediastinum[tiab] OR "mediastinal emphysema"[tiab] OR "subcutaneous emphysema"[tiab] OR pneumoperitoneum[tiab] OR barotrauma[tiab] OR "pleural injury"[tiab] OR "pleural breach"[tiab] OR "pleural tear"[tiab] OR "thoracic complication*"[tiab])<br><br>("Postoperative Complications"[Mesh] OR "Intraoperative Complications"[Mesh] OR "Iatrogenic Disease"[Mesh] OR postoperative[tiab] OR intraoperative[tiab] OR perioperative[tiab] OR iatrogenic[tiab] OR complication*[tiab])<br><br>Final: #1 AND #2 AND #3

Database	Strings
	Filters: Humans. No date, language or article-type limit.
Medline-Ovid	(exp Mammaplasty/ or (autologous breast reconstruct* or free flap breast reconstruct* or free-flap breast reconstruct* or microvascular breast reconstruct* or DIEP or deep inferior epigastric perforator or TRAM or transverse rectus abdominis myocutaneous or muscle sparing TRAM or muscle-sparing TRAM or SIEA or superficial inferior epigastric artery or latissimus dorsi flap* or latissimus dorsi breast reconstruct*).ti,ab,kf.) and (exp Pneumothorax/ or exp Mediastinal Emphysema/ or (pneumothorax* or pneumomediastinum* or mediastinal emphysema or pleural injur* or pleural breach* or pleural tear* or pleural perforat* or barotrauma or thoracic complication* or thoracic air complication*).ti,ab,kf.)
Wiley Online Library	("breast reconstruction" OR "DIEP" OR "TRAM" OR "latissimus dorsi" OR "free flap" OR mammaplasty OR mastectomy)<br>AND<br>(pneumothorax OR pneumomediastinum OR "pleural injury" OR barotrauma OR "subcutaneous emphysema")
